# The Hypocholesterolemic Potential of the Edible Algae *Fucus vesiculosus*: Proteomic and Quantitative PCR Analysis

**DOI:** 10.3390/foods12142758

**Published:** 2023-07-20

**Authors:** Rebeca André, Rita Pacheco, Ana Catarina Alves, Hugo M. Santos, Mafalda Bourbon, Maria Luísa Serralheiro

**Affiliations:** 1 BioISI—Biosystems & Integrative Sciences Institute, Faculdade de Ciências, Universidade de Lisboa, 1749-016 Lisboa, Portugal; catarina.alves@insa.min-saude.pt (A.C.A.); mafalda.bourbon@insa.min-saude.pt (M.B.); mlserralheiro@fc.ul.pt (M.L.S.); 2 Department of Chemical Engineering, ISEL—Instituto Superior de Engenharia de Lisboa, Rua Conselheiro Emídio Navarro, 1, 1959-007 Lisboa, Portugal; 3 Centro de Química Estrutural, Institute of Molecular Sciences, Universidade de Lisboa, 1749-016 Lisboa, Portugal; 4 Unidade de I&D, Grupo de Investigação Cardiovascular, Departamento de Promoção da Saúde e Prevenção de Doenças Não Transmissíveis, Instituto Nacional de Saúde Doutor Ricardo Jorge, 1649-016 Lisboa, Portugal; 5 LAQV@REQUIMTE, Department of Chemistry, NOVA School of Science and Technology, Universidade NOVA de Lisboa, 2829-516 Caparica, Portugal; hms14862@fct.unl.pt; 6 PROTEOMASS Scientific Society, Madan Park, Rúa dos Inventores, 2825-182 Caparica, Portugal; 7 Department of Chemistry and Biochemistry, Faculdade de Ciências, Universidade de Lisboa, Campo Grande, C8 Bldg, 1749-016 Lisboa, Portugal

**Keywords:** *Fucus vesiculosus*, cholesterol synthesis, cholesterol excretion, ezetimibe, NPC1L1, ABCG5

## Abstract

A brown seaweed consumed worldwide, *Fucus vesiculosus*, has been used to prevent atherosclerosis and hypercholesterolemia, among other uses. However, the mechanisms of action that lead to these effects are not yet fully understood. This work aims to study the in vitro effect of an aqueous extract of *F. vesiculosus*, previously characterized as rich in phlorotannins and peptides, on the expression of different proteins involved in the synthesis and transport of cholesterol. A proteomic analysis, Western blot, and qRT-PCR analysis were performed to identify protein changes in HepG2 cells exposed to 0.25 mg/mL of the *F. vesiculosus* extract for 24 h. The proteomic results demonstrated that, in liver cells, the extract decreases the expression of four proteins involved in the cholesterol biosynthesis process (CYP51A1, DHCR24, HMGCS1 and HSD17B7). Additionally, a 12.76% and 18.40% decrease in the expression of two important transporters proteins of cholesterol, NPC1L1 and ABCG5, respectively, was also observed, as well as a 30% decrease in NPC1L1 mRNA levels in the cells exposed to the extract compared to control cells. Our study reveals some of the mechanisms underlying the actions of bioactive compounds from *F. vesiculosus* that may explain its previously reported hypocholesterolemic effect, future prospecting its use as a functional food.

## 1. Introduction

The principal cause of global mortality is cardiovascular diseases (CVDs), specifically ischemic heart disease (IHD) and stroke [1]. The prevalence of CVDs is linked to unhealthy dietary habits with high consumption of salt, refined carbohydrates and fats, such as cholesterol [2,3]. High levels of cholesterol in blood and tissues are one of the major risks for lethal myocardial infarction and stroke due to the formation of arterial plaques [4]. It is estimated that between 1990 and 2017 the global number of deaths increased by approximately 910,000 due to high levels of non-HDL cholesterol which consequently led to IHD and stroke [5].

Previous studies report that the treatment of high cholesterol levels leads to significant health benefits [1]. The plasma cholesterol levels can be regulated through different mechanisms, such as the de novo cholesterol synthesis, synthesis of bile acids, excretion of cholesterol to bile and intestinal cholesterol absorption [2].

Cholesterol synthesis in liver is a complex, multi-step process involving different enzymes. There are two main possible pathways for cholesterol synthesis the Bloch and the Kandutsch–Russell pathway [6,7]. Both pathways share the initial steps from acetyl-CoA to lanosterol synthesis. However, after this stage, they diverge, although they do have a few enzymes in common [6,7].

Reverse cholesterol transport (RCT) is an important a pathway that transports cholesterol from non-hepatic tissues to the liver for secretion in bile. Although the transport of cholesterol to phospholipids acceptors occurs spontaneously in all cells, this is an inefficient process by itself [8]. In RCT, three important proteins that play a key role in cholesterol transport are known, namely ATP binding cassette transporter (ABC) A1, ABCG1 and scavenger receptor BI (SR-BI). Cholesterol can also be reabsorbed by the liver by Niemann-Pick C1-Like 1 (NPC1L1) protein, an essential protein that regulates plasma cholesterol levels [2]. In the liver, NPC1L1 has the ability to transport free cholesterol from the canalicular bile back to hepatocytes, thus presenting an opposite mechanism to the ABCG5/ABCG8 transporter which regulates biliary cholesterol secretion [9]. This protein is also critical for regulating intestinal cholesterol absorption [2]. The NPC1L1 protein is a molecular target for one of the most used drugs in treating hypercholesterolemia, ezetimibe [10]. This drug acts by blocking the internalization of NPC1L1 and, consequently, decreasing cholesterol uptake [11], leading to the inhibition of both intestinal absorption of dietary cholesterol and biliary cholesterol absorption [12]. To regulate blood cholesterol levels, the American Heart Association (AHA) and the American College of Cardiology (ACC) guidelines also suggest adopting a healthy dietary pattern. This includes consuming foods low in saturated fat and nutrients from natural sources [13]. These recommendations remain current, despite epidemiological data, meta-analyses and clinical interventions indicating that there is no direct correlation between cholesterol intake and blood cholesterol levels, though it is important to note that consuming cholesterol sources with saturated and trans fats increases plasma cholesterol [14].

In recent years, algae have received attention as a food product due to their beneficial effects, namely brown algae, which are rich in several bioactive compounds as phlorotannins [15]. *Fucus vesiculosus* is a brown algae consumed in different parts of the world and traditionally used for several reported beneficial effects, including the prevention of mineral deficit, weight loss, arthrosis, arthritis, atherosclerosis, viscous blood and hypercholesterolemia, and as an adjuvant for menopause [16,17,18,19]. Particularly regarding the reduction of cholesterol levels, in vivo and in vitro studies with extracts rich in phlorotannins have reported decreases in LDL, triglycerides and total cholesterol levels [20,21,22]. However, the mechanisms of action of brown algae biomolecules that lead to cholesterol-lowering effects remain unclear. Our group has already reported an in vitro study with the aqueous extract of *F. vesiculosus*, prepared in the form of soup and characterized as rich in phlorotannins and peptides, demonstrating its beneficial effect in vitro by inhibiting the intestinal absorption of cholesterol and its synthesis [23]. Another published study demonstrated that the extract under study, in vitro, led to an increase in several lipid compounds in HepG2 cells, including fatty acid amides, which are described as inhibitors of the ACAT enzyme and consequently inhibitors of cholesterol absorption and plasma cholesterol levels [24].

The objective of the present work is to evaluate the effect of the aqueous extract of *F. vesiculosus*, prepared as soup and previously characterized as rich in phlorotannins and peptides [23], on the liver proteins involved in the cholesterol biosynthetic process, through proteomic analysis. It is also aimed to study the specific effect of *F. vesiculosus* extract on important cholesterol transporter proteins NPC1L1 and ABCG5, using molecular assays as qRT-PCR and Western Blot.

## 2. Materials and Methods

### 2.1. Chemicals

All chemicals were of analytical grade. Water, methanol (MeOH), formic acid and acetonitrile LC-MS grade Optima were purchased from Fisher Scientific (Waltham, MA, USA). Ethanol 96% was bought from Carlo Erba (Peypin, France). Dulbecco’s modified Eagle medium (DEMEM), Trypsin, Glutamine, Phosphate-Buffered Saline (PBS), fetal bovine serum (FBS) and Tween 20 were obtained from Lonza^®^ (Verviers, Belgium). Glacial acetic acid and Tris(hydroxymethyl)aminomethane were obtained from Merck Milipore^®^ (Burlington, MA, USA).

Glycine, Bovine Serum Albumin (BSA), Igepal^®^ CA-630, Iodoacetamide, Urea, Ammonium bicarbonate and Glucose were purchased from Sigma-Aldrich (Barcelona, Spain). Pierce™ DTT (Dithiothreitol), Pierce™ Trypsin Protease MS Grade, mini Protein Gel NuPAGE™ 4 to 12% Bis-Tris, Bolt^®^ MOPS Transfer Buffer (20×), Bolt^®^ MOPS SDS Running Buffer (20×), PageRuler™ Prestained Protein Ladder and 4× Bolt™ LDS Sample Buffer were obtained from Thermo Fisher Scientific (Waltham, MA, USA). Coomassie Brilliant Blue R-250 was purchased from BIORAD^®^ (Berkeley, CA, USA). NZYBlue Protein Marker, 5× SDS-PAGE Sample Loading Buffer, NZY Total RNA Isolation kit, NZY First-Strand cDNA Synthesis Kit and NZYSpeedy qPCR Green Master Mix ROX plus were purchased from Nzytech^®^ (Lumiar, Portugal). AmershamTM ProtranTM Premium 0.45 µm Nitrocellulose Blotting Membrane, AmershamTM ECLTM Prime Western Blotting Detection Reagents, AmershamTM ECLTM Prime Blocking Agent and Amersham ECL™ anti-rabbit IgG, horseradish peroxidase-linked whole antibody (from donkey) were obtained from VWR International (Radnor, PL, USA). ABCG5 and NPC1L1 antibodies were purchased from Novus Biologicals. NPC1L1 and GAPDH primers were purchased from Alfagene (Lisbon, Portugal).

### 2.2. Algae Extract Preparation and Identification of Compounds

Whole dried brown seaweed *F. vesiculosus* Linnaeus from the North Atlantic Ocean was purchased from Celeiro diet., Lisbon, Portugal (imported by Américo Duarte Paixão Lda, Lot number 03ALG2731901). The aqueous extraction and the purification by Solid Phase Extraction (SPE) of *F. vesiculosus* were performed as described in [23]. The identification of the extract compounds was performed though Liquid Chromatography by High Resolution Mass Spectrometry (LC-HRMS/MS) using an Elute OLE UHPLC system interfaced with a quadrupole time-of-flight (QqToF) Impact II mass spectrometer equipped with an electrospray source (ESI) (Bruker DaltoniK GmbH, Bremen, Germany). The results were previously presented [25].

### 2.3. Cell Culturing

Caco-2 cells (ECACC 86010202), a human colorectal adenocarcinoma epithelial cell line, and HepG2 (ECACC 85011430), a human hepatocellular liver carcinoma cell line, were cultured in DMEM supplemented with 10% and 20% FBS, respectively, and 2 mM L-glutamine at 37 °C in an atmosphere with 5% CO_2_. The culture cells were kept in sub-confluence with trypsinization every 72 h.

### 2.4. Membrane Protein Extraction and SDS-PAGE Electrophoresis

HepG2 cells seeded in T75 flasks were under contact with the *F. vesiculosus* extract at 0.25 mg/mL (IC_30_) [24] and culture medium without FBS (control) for 24 h. After incubation time, the cells were washed twice with PBS, scraped with water and transferred to a pre-weight eppendorf tube. The cells were then lyophilized in a Heto PowerDry 3000 apparatus (Thermo Fisher Scientific). Approximately 3 mg of cells of control and cells exposed to extract was used to obtain the fractions of membrane proteins using the Mem-PER Plus Membrane Protein Extraction Kit (Thermo Scientific™) following the manufacturer’s indications. The different samples of both protein fractions were separated under reducing conditions in NuPAGE 4 to 12% gradient gels (Invitrogen™, Waltham, MA, USA) using a Mini Gel Tank (Invitrogen™) according to the manufacturer’s instructions. The gels were stained with 40% Coomassie R-250 blue, 50% methanol and 10% glacial acetic acid for 1 h and distaining with a solution of 7.5% glacial acetic acid, 10% ethanol and 82.5% distilled water overnight. Gels were photographed using ImageQuant LAS 50 (GE Healthcare Life Sciences^®^, Chicago, IL, USA), and the images were analyzed using ImageJ 1.47 software.

### 2.5. In-Gel Protein Digestion, Nano-LC−ESI−MS/MS and Data Analysis

In-gel protein digestion was performed as described in [25]. The nLC-MS/MS analysis of the resulting peptide digests was performed as described in [26], using an Ultimate 3000 nLC apparatus coupled to a UHR-QqTOF IMPACT HD apparatus (Bruker Daltonics, Bremen, Germany) with a CaptiveSpray ion source (Bruker Daltonics, Bremen, Germany). Raw LC−MS/MS data were processed in MaxQuant (V.1.6.10.43) for automated protein identification. MS raw files were analyzed by MaxQuant software, version 1.6.10.43 [27], and peptide lists were searched against the human Uniprot FASTA database. A contaminant database generated by the Andromeda search engine [28] was configured with cysteine carbamidomethylation as a fixed modification and N-terminal acetylation and methionine oxidation as variable modifications. We set the false discovery rate (FDR) to 0.01 for protein and peptide levels with a minimum length of seven amino acids for peptides, and the FDR was determined by searching a reverse database. Enzyme specificity was set as C terminal to arginine and lysine as expected using trypsin. A maximum of two missed cleavages were allowed. Data processing was performed using Perseus (version 1.6.2.3) with default settings [29].

All proteins and peptides matching the reversed database were filtered out. Subcellular localization and gene ontology analysis were performed using STRING online resources at https://string-db.org/ (accessed on 21 May 2022) and ClueGo plug-in in Cytoscape (V3.9.0), respectively [30].

### 2.6. Western Blot Analysis

HepG2 cells were seeded in T25 culture flasks and after confluence were exposed to DMEM medium without FBS containing 0.25 mg/mL of *F. vesiculosus* aqueous extract purified by SPE (*F. vesiculosus* extract). After 24 h, the cells were scraped and collected with water and lyophilized. The cells were dissolved in lysis buffer (Igepal 4%, DTT 1%, Urea 6 M), at a concentration of 0.025 mg of cell/mL, followed by sonication, for 5 min, and centrifugation for 10 min at 10,000 rpm. The cells precipitated were used for Western blot following the protocol described in [31]. The assays were performed in triplicate, and the results are presented in terms of mean and standard deviation.

### 2.7. Real Time Quantitative PCR

HepG2 cells were seeded in T75 culture flasks and after confluence were exposed to DEMEM medium without FBS, to 0.25 mg/mL of *F. Vesiculosus* aqueous extract and to 100 µM of ezetimibe. At 24 h post-exposition, RNA was harvested from cells using NZY First-Strand cDNA Synthesis Kit. Each experiment of RNA extraction was carried out with duplicate samples. cDNA was synthesized from 1 µg of RNA using NZY First-Strand cDNA Synthesis Kit following the manufacturer’s protocol. The qRT-PCR was performed in triplicates using NZYSpeedy qPCR Green Master Mix (2×), ROX plus. The primers sequence used to amplify GAPDH and NPC1L1 genes were described in [32]. Reactions were performed in the Applied Biosystems 7500 Real-Time PCR System (Thermo Fisher Scientific), and the real-time PCR program consisted of 40 cycles (95 °C for 15 s and 62 °C for 30 s) after an initial 10 min incubation at 95 °C. The expression of NPC1L1 was determined relative to GAPDH, and data are presented as mean values with standard deviations.

### 2.8. Statistical Analysis

The data were expressed as mean ± standard deviation using the Microsoft^®^ Excel 2016 software. Statistical analysis was performed using one-way analysis of variance (ANOVA) using software developed by Microsoft^®^ (Microsoft office 365, Washington, DC, USA) with a *p*-value ≤ 0.05 considered as significant.

## 3. Results and Discussion

### 3.1. Effect of F. vesiculosus on Hepatic Proteins

**Figure 1:**
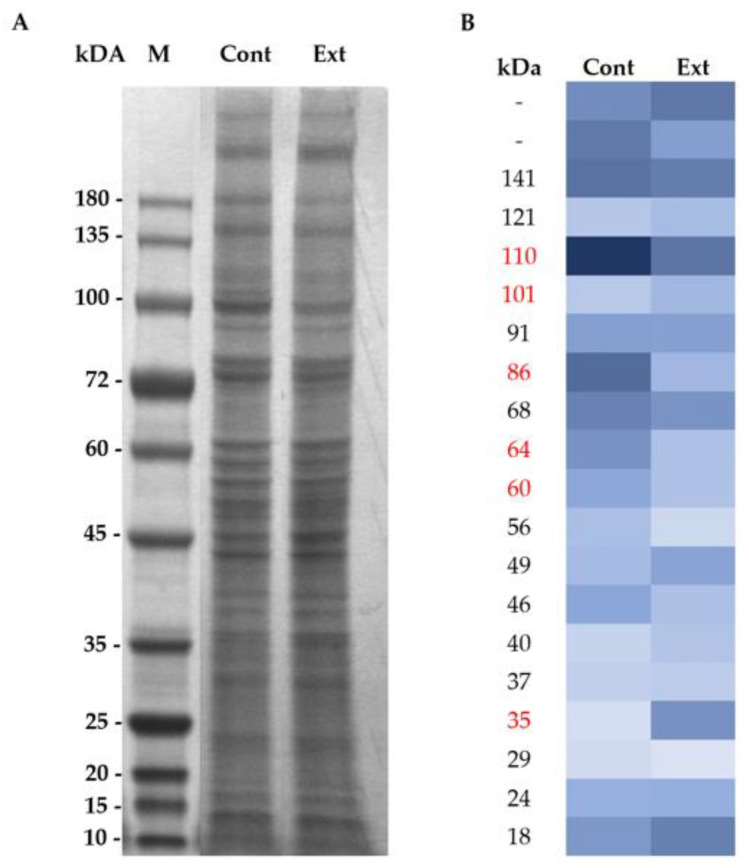
Effect of *F. vesiculosus* aqueous extract on soluble membrane proteins of HepG2. (**A**)—Gel obtained from SDS-PAGE of the soluble membrane protein fraction of HepG2 cells under the effect of: (Cont), cell culture media and (Ext), 0.25 mg/mL of *F. vesiculosus* extract; (M), marker proteins. (**B**)—Heat map generated from SDS-PAGE gel analyze with Image J software reflecting the protein band intensity of soluble membrane protein fraction of HepG2 cells exposed to (Cont) cell culture media and (Ext) *F. vesiculosus* extract. Cells samples are arranged in columns, protein band intensity, and estimated molecular weight in rows. Dark blue shades correspond to a high-intensity protein band; light blue shades correspond to a low-intensity protein band.

Different studies have been carried out on the hypocholesterolemic effect of different brown seaweeds. However, the molecular mechanisms of the bioactive compounds in brown seaweeds that contribute to a decrease in total cholesterol levels are still not fully understood. In this study, the effect of *F. vesiculosus* extract on hepatic proteins was evaluated by SDS-PAGE of membrane proteins from HepG2 cells exposed to a non-cytotoxic concentration of *F. vesiculosus* extract [24] (Figure 1).

The extract caused several changes in the relative intensity of different proteins (Figure 1A,B). Some 65% of the bands analyzed using the ImageJ program showed significant changes (ANOVA test with *p*-value ≥ 0.05) in their intensity in the presence of the extract compared to the control (Figure 1B).

#### Proteomic Analysis

**Figure 2:**
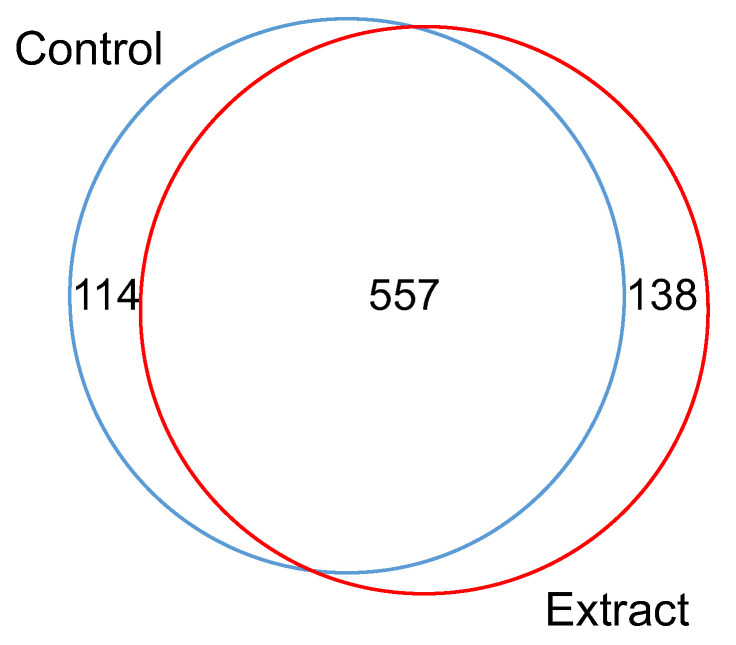
Venn diagram showing the differentially expressed proteins from comparative proteomic analysis of HepG2 cells control and cells exposed to 0.25 mg/mL of *F. vesiculosus* (http://bioinformatics.psb.ugent.be/webtools/Venn/ (accessed on 20 May 2022)).

A proteomic analysis was conducted to identify the HepG2 cells proteins present in the previous SDS-PAGE. For the proteomic assay, gel bands from distinct zones of the gel with different molecular weights (highlighted in red in Figure 1B) and different intensities were removed from both control and cell under the effect of the extract. A total of 809 protein groups were identified, from which 671 were detected in the control experiments and 695 were detected in the extract. Some 68.9% (557) of the protein groups was detected in both control and test cells, whereas 114 proteins were exclusively present in the control cells, and 138 were exclusively present in cells exposed to the extract (Figure 2).

**Figure 3:**
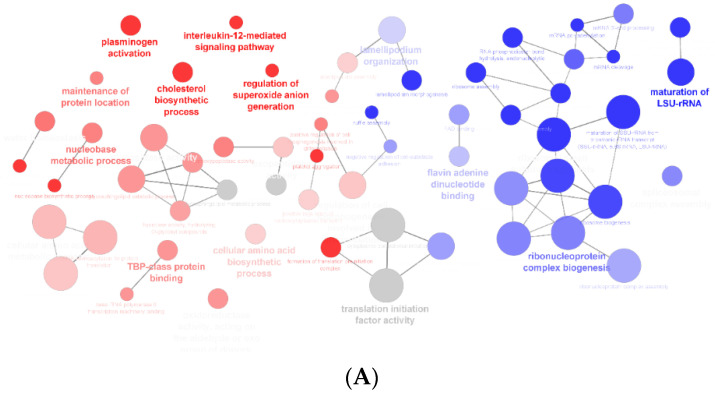

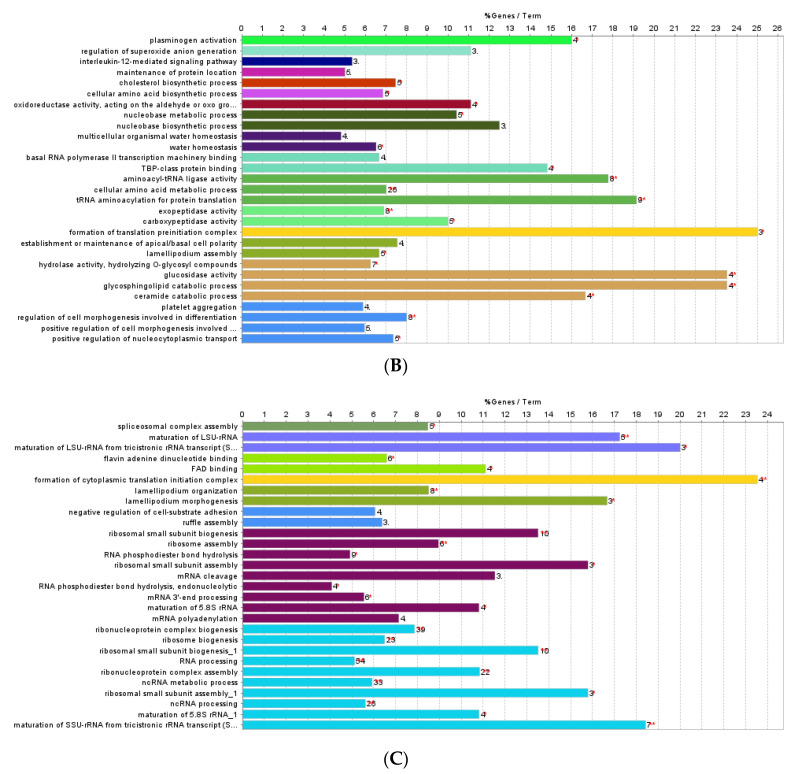
(**A**)—Networking of ClueGO analysis of significant enrichment GO biological process and molecular function (term *p*-value corrected with Bonferroni step down, *p* < 0.05), which represent the term enrichment of proteins from HepG2 control cells (red), proteins from HepG2 cells exposed to *F. vesiculosus* (blue) and terms from both clusters (gray). (**B**)—Bar chart representing the percentage of gene by term of GO Biological process and GO molecular function from HepG2 control cells and HepG2 exposed to *F. vesiculosus* (**C**). The red asterisk indicate that protein/gene can be part of multiple biological processes. The number next to * indicates how many additional term occurrences exist in other gene ontology term.

Proteins expressed exclusively in the control group and those expressed exclusively in cells exposed to the extract were analyzed using different databases, depending on the specific objectives intended with the analysis of the results. The analysis of gene ontology (GO) terms biological process (BP) and molecular function (MP) were analyzed using ClueGO Cytoscape App. The networks in Figure 3A show the GO terms more significantly enriched for control cells (red) and cells exposed to the extract (blue), followed by the statistics graphs representing the percentage of gene by term for the control cells (Figure 3B) and for the cells exposed to extract (Figure 3C). By analysis of Figure 3, it is evident that proteins from both groups are involved in various biological processes and exhibit different molecular functions. However, in this study, our focus was to highlight the proteins associated with the cholesterol biosynthetic process, considering the previously reported hypocholesterolemic effect of the *F. vesiculosus* aqueous extract under investigation [23,24].

**Figure 4:**
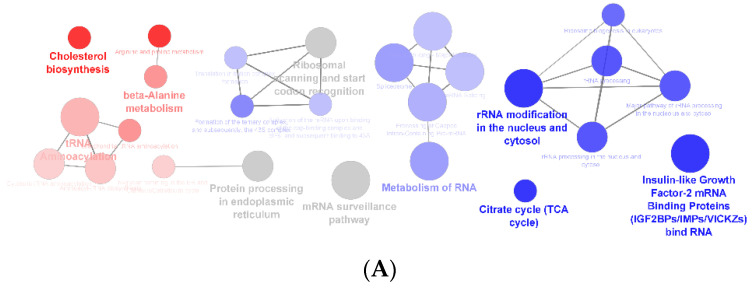

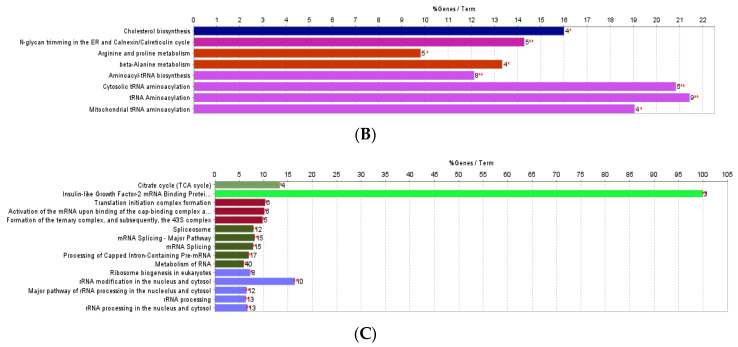
(**A**)—Networking of ClueGO significant enrichment pathways analysis from KEEG and Reactome (term *p*-value corrected with Bonferroni step down, *p* < 0.05), which represent the terms enrichment of proteins from HepG2 control cells (red), HepG2 cells exposed to *F. vesiculosus* (blue) and terms from both clusters (gray). (**B**)—Bar chart representing the percentage of gene by term of each enriched pathway from HepG2 control cells (**B**) and HepG2 exposed to *F. vesiculosus* (**C**). The red asterisk indicate that protein/gene can be part of multiple biological processes. The number next to * indicates how many additional term occurrences exist in other gene ontology term.

The KEGG and Reactome pathways in which the proteins under study participate were also analyzed using ClueGO Cytoscape App (Figure 4).

**Figure 5:**
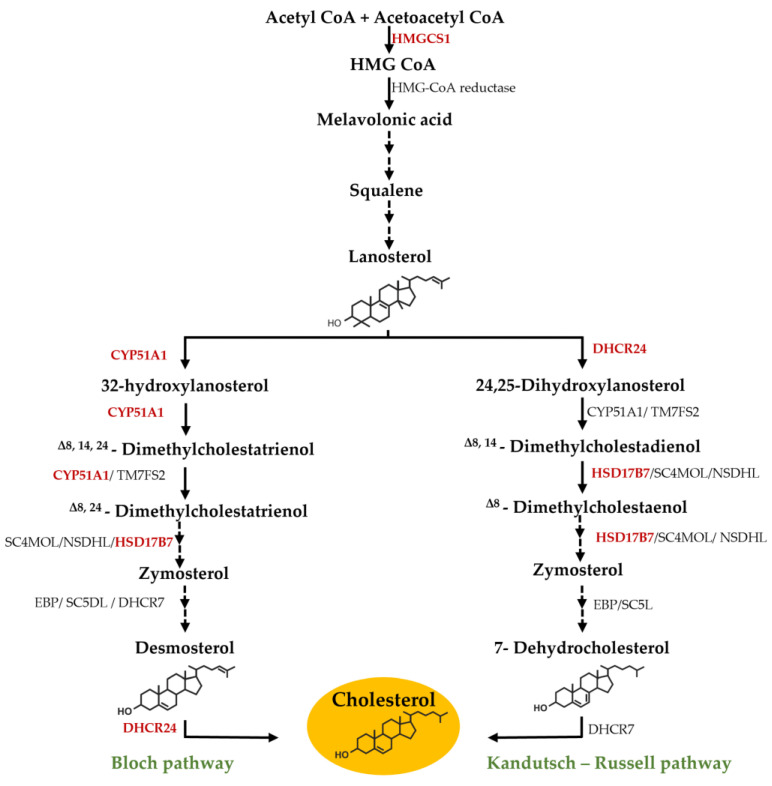
Cholesterol synthesis pathway. The pathway proteins that proteomic analysis revealed to be present just in HepG2 control cells and not in HepG2 cells exposed to the extract are highlighted in red.

The protein Lanosterol 14-alpha demethylase (CYP51A1), enzyme 3-hydroxysterol 24-reductase (DHCR24), 3-Hydroxy-3-Methylglutaryl-CoA Synthase 1 (HMGCS1) and Hydroxysteroid 17-Beta Dehydrogenase 7 (HSD17B7) involved in the cholesterol biosynthesis pathway (Figure 5) were detected in the control cells, whereas they were not detected in the cells after 24 h contact with the *F. vesiculosus* extract.

DHCR24 catalyzes the final step of the Bloch pathway of cholesterol synthesis and also catalyzes the first step of Kandutsch–Russell pathway [7], as shown in Figure 5. Previous studies with HepG2 cells demonstrated that the inhibition of DHCR24 leads to decreased cholesterol production [33]. HSD17B7 is another enzyme common to both pathways of cholesterol synthesis (Figure 5). A previous study demonstrated that the lack of this enzyme inhibits the de novo cholesterol biosynthesis [34]. CYP51A1 is known as a critical cholesterologenic enzyme essential for regulating cholesterol biosynthesis [35]. In previous studies, inhibition of CYP51A1 indicated significant reductions in serum levels of total cholesterol and serum low-density proteins [35]. Furthermore, inhibition of CYP51A1 leads to accumulation of lanosterol which, in turn, induces the degradation and ubiquitination of HMG CoA reductase enzyme (HMGR), the rate-limiting step in cholesterol synthesis [35,36]. In our previous study, *F. vesiculosus* extract also showed a significant capacity to inhibit in vitro the HMGR with an half inhibitory concentration (IC_50_) of 4.16 µg/mL [23]. The other protein from the cholesterol biosynthesis pathway, HMGCS1, catalyzes the condensation of acetyl-CoA with acetoacetyl-CoA to form HMG-CoA (Figure 5) (https://www.uniprot.org/uniprot/Q01581 (accessed on 30 May 2022)), and its inhibition consequently leads to inhibition of cholesterol synthesis [37].

As mentioned, these four proteins were only detected in control cells, which means that the presence of the extract is leading to a decrease in the expression of these proteins, leading in turn to the inhibition of cholesterol synthesis. The description of these molecular mechanisms of action of the extract on proteins involved in the synthesis of cholesterol is novel, which is in line with the hypocholesterolemic effect already reported [18].

### 3.2. Effect of Fucus vesiculosus on Cholesterol Transporters

#### *F. vesiculosus* Aqueous Extract Decrease Hepatic Expression of NPC1L1 and ABCG5

A Western blotting and qRT-PCR techniques were performed to study the specific effect of *F. vesiculosus* extract on the hepatic expression of two important cholesterol transporter proteins, NPC1L1 and ABCG5.

This extract was previously characterized through LC-HRMS/MS analysis, and the results demonstrated that phlorotannin derivatives and small peptides represented 94% of the intensities detected. As the extract under study is rich in phenolic compounds [23], namely phlorotannins, the Western blot technique was performed using the same weight of cells per sample [31]. The standard protocol for this technique is based on standardizing the samples-on protein amount; however, previous studies [31,38] have reported that different phenolic compounds affect cellular protein content, making it impossible to use the total protein content and internal control proteins to do the standardizing of samples.

**Figure 6:**
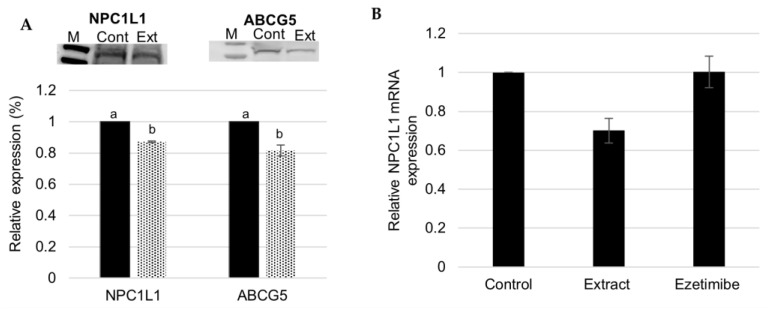
(**A**) Western blot results representing the effect of *F. vesiculosus* aqueous extract (0.25 mg/mL) on the protein expression of NPC1L1 and ABCG5 in HepG2 cells using the same weight of cells (0.025 mg of cell/mL of lysis buffer), (**B**) relative NPC1L1 mRNA expression in HepG2 under the effect of *F. vesiculosus* aqueous extract (0.25 mg/mL) and ezetimibe (100 µM) determined by qRT-PCR (normalized to GAPDH). Different superscript letters (a–b) correspond to values of relative expression for each gene that can be considered statistically different (*p* ≤ 0.05).

Figure 6A shows that, in HepG2 cells, the extract led to a 12.76 ± 0.47% decrease in NPC1L1 expression. The qRT-PCR demonstrated that the extract also led to approximately 30% inhibition of NPC1L1 mRNA. (Figure 6).

Previous studies have reported that overexpression of NPC1L1 in the liver causes an inhibitory effect on biliary cholesterol secretion, once it re-absorbs cholesterol from bile leading to high levels of liver cholesterol, and thus there is a risk of developing atherosclerosis and other CVDs [2,39]. Consequently, the overexpression of hepatic NPC1L1 may aggravate diet-induced atherosclerosis [2]. The *F. vesiculosus* extract effect on the inhibition of NPC1L1 mRNA and protein expression could, therefore, be beneficial by increasing biliary excretion of cholesterol and, consequently, contribute to decrease the risk of atherosclerosis.

One of the drugs most used for the therapy of hypercholesterolemia is ezetimibe, which acts to block NPC1L1 internalization and consequently decrease cholesterol uptake [11]. Previous studies also reported that ezetimibe treatment does not affects protein or mRNA expression of NPC1L1 [40]. Considering that the extract was seen to inhibit the expression of both NPC1L1 mRNA and protein, the study here reported could be a starting point for the search for new therapeutic strategy showing a different mechanism of action than ezetimibe.

*F. vesiculosus* aqueous extract also decreased the hepatic expression of ABCG5 by 18.40 ± 3.53% (Figure 6A) relative to the control cells. In the liver, the ABCG5 and the ABCG8 transporter form a complex (ABCG5/ABCG8) that mediates the cholesterol secretion to bile [39]. Based on this knowledge, one would expect the extract to induce hepatic expression of ABCG5 to promote hepatic excretion of cholesterol in the bile, but this was not observed. We hypothesize that, as the extract leads to inhibition of cholesterol synthesis, as demonstrated in the previous results presented in Section 3.2 and as observed in a previous in vitro study [23], there is less need to eliminate cholesterol from liver into the bile, leading, consequently, to a lower expression of the ABCG5 protein. The observed inhibition of NPC1L1 expression also leads to lower cholesterol levels in the liver which also leads to a lesser need to eliminate cholesterol from this organ into the bile.

The effect of statins, another group of drugs often prescribed to lower cholesterol levels, in the expression of the ABCG5/ABCG8 transporter is still an area under study. Statins primarily act by inhibiting HMGR leading to cholesterol synthesis inhibition. Studies in vivo demonstrated that some statins increase the expression of ABCG5 leading to increased biliary excretion of cholesterol [41,42]. By contrast, other studies reported that atorvastatin and pravastatin decrease the expression of ABCG5 at the intestinal and hepatic level [40,41].

Further in vivo studies and with primary cells are needed to clarify the effect of *F. vesiculosus* extract on biliary cholesterol excretion and on blood and liver cholesterol levels and to better understand its inhibitory effect towards ABCG5 protein expression.

## 4. Conclusions

This study describes the mechanisms of action on liver proteins of *Fucus vesiculosus,* rich in phlorotannins and peptides, supporting its previously described hypocholesterolemic effect.

In this work, it is reported that the aqueous extract of *F. vesiculosus* inhibits the expression of four proteins involved in cholesterol synthesis, CYP51A1, DHCR24, HMGCS1 and HSD17B7, and therefore this effect may impact the cholesterol synthesis pathway. Also, it was seen that the extract has the capacity to decrease an important cholesterol transporter NPC1L1 protein expression, along with its mRNA expression. This type of effect has the potential to increase the biliary excretion of cholesterol, thereby potentially lowering circulating cholesterol levels, a factor that reduces the risk of atherosclerosis.

These findings suggest that the extract may act both on cholesterol synthesis and excretion, yet by different action mechanisms compared to the drugs often prescribed to treat hypercholesterolemia, statins and ezetimibe. As stated, the first targets HMGR enzyme inhibiting cholesterol liver biosynthesis, and the latter is used to regulate the intestinal/hepatic levels of cholesterol absorption. While statins may be associated with secondary effects and ezetimibe is not highly efficient, often requiring combination therapy with statins, the observed hypocholesterolemic results of the extract indicate promising avenues for future research aimed at the treatment and prevention of hypercholesterolemia.

Furthermore, in addition to its inhibitory effects on cholesterol synthesis and NPC1L1expression, the extract was also demonstrated to be able to reduce the expression of the cholesterol transporter protein ABCG5 in cells exposed to it. This observation suggests its potential also to reduce cholesterol export from the liver to the circulation.

Considering all these factors and the fact that *F. vesiculosus* is a natural food product that demonstrated such cooperative effects, this study provides strong scientific support for the utilization of this extract in the development of a supplement or a functional food with a hypocholesterolemic effect.

## Data Availability

Data is contained within the article.
